# The relationship between alcohol consumption, perceived stress, and CRHR1 genotype on the hypothalamic–pituitary–adrenal axis in rural African Americans

**DOI:** 10.3389/fpsyg.2015.00832

**Published:** 2015-06-18

**Authors:** Ezemenari M. Obasi, Elizabeth A. Shirtcliff, Gene H. Brody, James MacKillop, Delishia M. Pittman, Lucia Cavanagh, Robert A. Philibert

**Affiliations:** ^1^Hwemudua Addictions and Health Disparities Laboratory, Department of Psychological, Health, and Learning Sciences, University of Houston, Houston, TX, USA; ^2^Human Development and Family Studies, Iowa State University, Ames, IA, USA; ^3^Center for Family Research, University of Georgia, Athens, GA, USA; ^4^Psychiatry and Behavioral Neuroscience, McMaster University, Hamilton, ON, Canada; ^5^Graduate School of Education and Human Development, George Washington University, Washington, DC, USA; ^6^Department of Psychiatry, University of Iowa, Iowa City, IA, USA

**Keywords:** African Americans, alcohol, HPA-axis, stress, CRHR1, health disparities

## Abstract

**Objective:** Rurally situated African Americans suffer from stress and drug-related health disparities. Unfortunately, research on potential mechanisms that underlie this public health problem have received limited focus in the scientific literature. This study investigated the effects of perceived stress, alcohol consumption, and genotype on the hypothalamic–pituitary–adrenal (HPA) Axis. **Methods:** A rural sample of African American emerging adults (*n* = 84) completed a battery of assessments and provided six samples of salivary cortisol at wakeup, 30 min post wakeup, 90 min post wakeup, 3:00 PM, 3:30 PM, and 4:30 PM. **Results:** Participants with a TT genotype of the CRHR1 (rs4792887) gene tended to produce the most basal cortisol throughout the day while participants with a CC genotype produced the least amount. Increased levels of perceived stress or alcohol consumption were associated with a blunted cortisol awakening response (CAR). Moreover, the CAR was obliterated for participants who reported both higher stress and alcohol consumption. **Conclusion:** Perceived stress and alcohol consumption had a deleterious effect on the HPA-Axis. Furthermore, genotype predicted level of cortisol production throughout the day. These findings support the need to further investigate the relationship between stress dysregulation, drug-use vulnerability, and associated health disparities that affect this community.

## Introduction

African Americans are disproportionately exposed to chronic stress by way of high levels of racism, discrimination, violence, crime, neighborhood disorganization, unemployment, financial strain, and low socioeconomic status (Clark et al., 1999; Dalaker, 2001; Centers for Disease Control and Prevention, 2004, 2005; Brody et al., 2015; Myers et al., 2015). Persistent exposure to chronic stress causes “wear-and-tear” on the body’s regulatory system and compromises its capacity to recover efficiently from incessant exposure to environmental stressors (McEwen, 1998). Consequent alterations in stress responsive systems is theorized to heighten drug-use vulnerability as drug use provides a coping strategy for alleviating negative affect that is becoming increasingly more difficult to manage and temporally normalizes stress physiology (Koob and Le Moal, 2001, 2008). Subsequently, drug abuse and dependency—compounded by a dysregulated stress system—places African Americans at-risk for disproportionate levels of drug use consequences associated with violence (e.g., homicide, suicide, child abuse, and domestic violence), injuries (e.g., crashes, falls, burns, and drowning), and health disparities (e.g., prostate cancer, liver cancer, chronic liver disease, hypertension, myocardial infarction, gastritis, pancreatitis, STDs, meningitis, and poor control of diabetes; Naimi et al., 2003; National Institute on Drug Abuse, 2003; Centers for Disease Control and Prevention, 2004). The purpose of this study was to investigate the effects of stress, alcohol consumption, and genotype on the hypothalamic–pituitary–adrenal (HPA) axis.

The body responds to internal and external stressors via the stress-response system or HPA-axis. Drug use has been linked to the stress-response system in both rats (Coste et al., 2000) and humans (Lovallo et al., 2000; Fox et al., 2007). Specifically, dysregulation of the HPA has been associated with drug dependence, withdrawal, and relapse (Koob and Le Moal, 2001; Lovallo, 2006). However, the underlying mechanisms for this link remain unclear (Sommer et al., 2008). Recent investigations have considered the role of the corticotropin-releasing hormone (CRH) system as a critical modulator of HPA stress reactivity. Of particular focus, is the CRH receptor 1 (CRHR1) genotype. Specifically, CRHR1 is a 7-transmembrane G-protein-coupled receptor that is expressed in high density in the cerebral cortex, cerebellum, hippocampus, amygdala, and pituitary; and peripherally in the skin, ovaries, testes, and adrenal gland (Binder and Nemeroff, 2010).

Corticotropin releasing hormone receptor 1 is thought to be the principal receptor mediating the stress response and is highly expressed in the anterior pituitary, neocortex, hippocampus, amygdala, and cerebellum (Timpl et al., 1998). Furthermore, it has been found to be associated with the pathophysiology of anxiety, including PTSD, depression, and heavy alcohol consumption (Treutlein et al., 2006; Bradley et al., 2008; Wasserman et al., 2008, 2009; Binder and Nemeroff, 2010; Amstadter et al., 2011; Enoch, 2011). It has also been found to modulate dopamine function, ethanol-induced enhancement of GABAergic synaptic transmission, and reward learning (Nie et al., 2004; Bogdan et al., 2011). CRHR1 knockout rat models have demonstrated that lacking CRHR1 significantly reduces the release of adrenocorticotropic hormone (ACTH) and corticosterone; resulting in an impaired stress response that cannot be compensated by any other system (Timpl et al., 1998). Extending these findings to human studies may illustrate how CRHR1 genotype could predict genetic vulnerabilities that have a direct bearing on the production of hormonal biomarkers in response to environmental demands.

Cortisol, a glucocorticoid, is a hormonal biomarker of HPA functioning (Frodl and O’Keane, 2013). Cortisol is produced in the adrenal cortex and has a relatively stable diurnal rhythm that is characterized by a sharp increase within 30 min of morning waking, followed by a steep decline by mid-morning, and gradual decline during the course of the day (Stone et al., 2001; Shirtcliff et al., 2012). This pattern is an important component of HPA regulation, as high morning levels help individuals prepare for the day’s events, and low evening levels permit critical immune and tissue repair (Dallman et al., 2002). Investigating this diurnal pattern provides a window into the extent to which the body’s stress response system is altered as a function of chronic stress (McEwen, 2004). Consistent with McEwen’s (2004) concept of allostasis, or longstanding changes in the set-points of the stress system, the individual is theorized as being capable of adapting or changing to meet the demands of a changing environment or social context. As the individual is consistently exposed to that environment over time, the inherent variability may become less malleable. Allostasis frames the interplay between genetic, neural, behavioral and environmental forces as a developmental phenomenon with social contextual cues shaping the phenotypic expression of the genetic and biological building blocks. A specific marker of the functioning of the HPA-axis is the cortisol awakening response (CAR).

It is estimated that the CAR represents a 40–75% increase in cortisol 20–45 min post waking (Wust et al., 2000; Chida and Steptoe, 2009; Fries et al., 2009; Zeiders et al., 2014). After the CAR, the morning portion of the diurnal rhythm is principally controlled by the anterior pituitary and is under strong genetic influence (Van Hulle et al., 2012). Individual differences in the CAR is thought to be a reliable indicator of HPA functioning specifically, and a correlate of psychological and physical health broadly (Chida and Steptoe, 2009; Gonzalez et al., 2009; Thorn et al., 2009; Zeiders et al., 2014). More specifically, a hyperactive CAR has been linked to experiences of general life stress, overload, and worrying (Wust et al., 2000; Chida and Steptoe, 2009; Fries et al., 2009). Furthermore, a hypoactive CAR has been linked to chronic stress, PTSD, fatigue, burnout, depression, and hopelessness (Chida and Steptoe, 2009; Fries et al., 2009). A blunted CAR has also been linked to poor health outcomes like cardiovascular, autoimmune, atopic, and psychiatric disease (Fries et al., 2009). Taken together, increases in CAR suggest a sense of arousal that is garnered to meet upcoming challenges while decreases in CAR may reflect a more dysregulated HPA as a function of chronic exposure to debilitating stress (Thorn et al., 2009).

Chronic activation of stress responsive systems by ongoing experiences of racism, violence, crime, unemployment, financial strain, and low-to-no socioeconomic status can cause “wear and tear” on regulatory systems by way of allostatic load (McEwen, 1998). Allostasis does not provide a simple prediction for unidirectional alterations, with both hypo- and hyper-arousal resulting from extreme environmental input (Gunnar and Vazquez, 2001). Salient stressful experiences may alter the “setpoint” for stress regulation along hypo- or hyper-arousal trajectories. A dysregulated stress system, in turn, may contribute to drug use and abuse (Koob and Le Moal, 2001).

The relationship between the dysregulation of the HPA-axis and drug addiction has led to mixed research findings. Previous research suggests that alcohol, cigarettes, and cocaine increase HPA activity (Adinoff et al., 1998, 2003; Goeders, 2002; Mendelson et al., 2005). Conversely, heroin addicts have been found to exhibit a hyporesponsive HPA, lower cortisol levels in basal condition, and reduced cortisol decrease in the evenings (Facchinetti et al., 1985; Kreek et al., 2005). Animal models suggest that sensitivity to stress, recovery from stress, and/or the uncontrollability of stressors, are strong predictors of drug use and abuse (Goeders and Guerin, 1994; Campbell and Carroll, 2001; Goeders, 2002). As the adrenal cortex begins to produce more cortisol, the individual may be more sensitized to use alcohol, potentially establishing a positive-feedback loop where cortisol and psychological stress increase motivation to use alcohol (Bernardy et al., 1996; Adinoff et al., 1998; Thayer et al., 2006).

To date, animal models have driven the direction of experimental research investigating the link between stress dysregulation and drug abuse. Rasmussen et al. (2001) found that chronic alcohol consumption produced neuroendocrine and behavioral responses in rats that may increase risk for future abuse. The activation of the HPA-axis and enhanced expression of CRH have been observed during acute phases of drug dependence withdrawal, including benzodiazepines, cannabis, cocaine, alcohol, and morphine in rats (Miller, 1997; Milanes et al., 1998). Taken together, there is a growing body of literature linking altered stress physiological functioning and drug use vulnerability.

This study was designed to investigate how chronic stress, alcohol consumption, and genotype affect HPA-axis functioning in a sample of rural African American emerging adults residing in the southeastern United States. Beyond the fact that this study includes an at-risk sample that is rarely included in this area of research, we are unaware of any study that includes indicators of environmental stress, alcohol consumption, and genotype as concurrent predictors of HPA functioning. It was hypothesized that increased levels of stress and alcohol consumption would have a larger effect on developing a blunted CAR in comparison to the individual contributions of stress or alcohol alone. The utility of focusing some of our investigation on the CAR is twofold. First, the CAR is a reliable indicator of HPA functioning with hyperactivity associated with the anticipation of acute stress and hypoactivity indicative of a dysregulated HPA in response to chronic exposure to unmitigated stress. Secondly, this biomarker may be a mechanism linking stress dysregulation to drug use vulnerability. Furthermore, we investigated if a person’s genotype of the CRH receptor site (CRHR1) would affect basal cortisol levels throughout the day.

## Materials and Methods

### Participants

Participants (*N* = 84) consisted of African Americans between the ages of 18 and 23 ($\bar{X}$ = 20.1, *SD* = 1.1). The majority of the participants were female (*n* = 49, 58.3%), unmarried (*n* = 81, 95.3%), and self-identified as 5th generation in response to immigrant status (*n* = 73, 85.9%). In response to highest level of education obtained, 4.8% had less than a high school education, 33.3% graduated from high school, 50% had some college or technical classes, 10.7% had a college degree, and 1.2% had some professional training. Moreover, 63.1% of the participants were currently unemployed.

### Procedures

Participants were recruited to participate in this study after completing their enrollment in the control condition of the adults in the making (AIM) project. This group represents a random sample of African Americans who reside in rural counties in the southeastern U.S. Following informed consent, participants were enrolled in this study and mailed a saliva collection kit. This collection kit contained all the necessary materials and instructions for collecting diurnal samples of salivary cortisol. Participants were instructed to provide six saliva samples in their home using the following schedule: (1) wakeup—before getting out of bed, (2) 30 min post wakeup, (3) 90 min post wakeup, (4) 3:00 PM, (5) 3:30 PM, and (6) 4:30 PM. Samples 1–3 were required as an assessment of the cortisol response to awakening. More specifically, sample 2 represents the cortisol awaking response (CAR). Samples 4–6 provided assessments of the diurnal down-regulation of the HPA-axis through the early evening on a typical day. Participants were instructed not to eat, drink, brush their teeth or smoke cigarettes 30 min prior to sample collection. Compliance with collection protocols were enhanced by having graduate research assistants (GRAs) review the protocol with the participants over the phone prior to starting the saliva collection process. The saliva collection kit also contained collection instructions and a collection diary for the purpose of modeling systematic variation (i.e., time) in our analyses. Female participants were asked to collect home saliva samples outside the menstrual cycle as hormones are uncharacteristically low during this time. Participants were also instructed to freeze the saliva samples in the provided kit immediately upon completion.

During the in-person assessment, two African American GRAs met the participants at a prearranged location (e.g., home) in the participant’s community. Participants were oriented to this study and asked to complete a battery of computerized assessments that were randomly administered using Medialab^™^ v2010 on a laptop PC. Upon completion, the participants completed a 90-day assessment of their alcohol consumption, provided the GRAs with the previously collected saliva samples, and were debriefed. The GRAs transported the frozen saliva samples to the laboratory in sealed coolers filled with freezer bricks in order to prevent a freeze-thaw cycle. Participants were compensated $25 for their participation in this study. This study was approved by the University of Georgia Institutional Review Board.

### Measures

#### Alcohol Consumption

Alcohol consumption over the past 90 days was measured using the Timeline Follow-Back method (Sobell and Sobell, 1992). Participants were provided with a 90-day calendar and asked to provide retrospective estimates of their daily substance use. Several memory aids (i.e., calendar, holidays, key dates, discrete events, and anchor points) were used to enhance recall and alcohol consumption was reported in standard drinks (i.e., 12 oz beer, 5 oz wine, 1 oz of hard liquor).

#### CRHR1

Participants provided DNA samples as part of their previous participation in the AIM project. Previously arrayed stock DNA was diluted to 2 ng/ul and robotically dispensed. An informative marker of the corticotropin releasing hormone receptor 1 (CRHR1) was identified from previous studies that investigated depression (Wasserman et al., 2008, 2009) and included a sample of African Americans (Bradley et al., 2008). The samples were amplified using primer probe sets and other reagents from Applied Biosystems (ABI Foster City, CA, USA), then genotyped using an ABI 7900 HT Sequence Detection System using our previously described protocol (Philibert et al., 2009). For the purpose of this study, we will focus on a CRHR1 single nucleotide polymorphism (SNP; dbSNP Marker: rs4792887; Cytogenetic Band: 17q21.31e). This gene encodes a G-protein that binds to neuropeptides of the CRH, a primary regulator of the HPA-Axis. This SNP did not deviate from Hardy-Weinberg equilibrium (*p* = 0.21) and had a 100% call rate in this sample.

#### Salivary Cortisol

All saliva samples were stored immediately in an ultracold laboratory freezer (–30°C), then shipped overnight frozen with dry-ice pellets to the Middleton Research Biodiagnostics Lab (Madison, WI, USA). On the day of assay, samples were thawed and cortisol was assayed in duplicate using a well-established enzyme-linked immunosorbent assay (ELISA) kit specifically designed for use with saliva (Salimetrics, State College, PA, USA). Mean intra-assay and inter-assay coefficients of variation (CVs) were 3.8% and 7.4%, respectively. Samples were reanalyzed if the CV for the duplicate measurements were >20%. Samples from the same individual were all assayed on the same run. To normalize distributions, extreme values of raw cortisol were winsorized.

#### Stress

Stress was measured by the Perceived Stress Scale (PSS; Cohen et al., 1983). The PSS is a 14-item self-report measure that assesses an individual’s perception of situations in their life that they deem stressful over the past month. The PSS is rated on a 5-point Likert scale ranging from “Never” (0) to “Very Often” (4). Summary scores for the PSS range between 0 and 56; with higher scores indicating more stress. Each item on the PSS assesses one’s perceived stress within the last month. In previous research, scores on the PSS demonstrated adequate internal consistency and test—retest reliability. The PSS produced scores with suboptimal but acceptable reliability in this sample (Cronbach’s *α* = 0.67).

For descriptive purposes, a stress card sort was used to measure eight potential stressors influencing one’s daily life: Family, Friends, Identity, Money, Neighborhood, Race, School, and Work. Each domain included common examples of potential stressors. Participants were instructed to rank order these stressors from the “Most Stressful” to “Least Stressful.” Next, they were asked to discuss what about their top stressor was so demanding to deal with.

### Analytic Strategy

Hierarchical linear models (HLMs) were use to estimate the trajectory of cortisol throughout the day and accurately account for the inherent nesting of saliva samples (*n* = 504; 6 samples per participant) at level 1 within participants (*n* = 84) at level 2. The dependent variable consisted of six samples of salivary cortisol measured at waking ($\bar{X}$_time_ = 9:24 AM; *TSW* = 0), 30 min after waking ($\bar{X}$_time_ = 9:55 AM, $\bar{X}$_TSW_ = 31.51 min, *SD* = 11.02), 90 min after waking ($\bar{X}$_time_ = 11:15 AM, $\bar{X}$_TSW_ = 110.80 min, *SD* = 61.58), 3:00 PM ($\bar{X}$ = 3:01 PM, *SD* = 22.20), 3:30 PM ($\bar{X}$ = 3:37 PM, *SD* = 21.13), and 4:30 PM ($\bar{X}$ = 4:25 PM, *SD* = 18.45). At level 1, two predictors of cortisol level accounted for the trajectory of cortisol throughout the day. First, time since waking (*β*_1 *TSW*_) was modeled in min and used to capture the passage of time associated with the collection of six saliva samples from waking to 4:30 PM. Assuming a diurnal slope of cortisol throughout the day, the slope of TSW would reflect a curvilinear decrease in cortisol from waking to 4:30 PM. However, it is likely that the participants would experience a peak cortisol response approximately 30 min after waking up. Therefore, we included a second variable to capture the (CAR; *β*_2 *CAR*_), which was modeled using a dummy variable that was coded 1 for Sample #2 (30 min after waking) and 0 for the remaining samples. Four samples were excluded from the HLM model since their sample was collected more than 50 min from waking. The intercept (*β*_0_) therefore represents cortisol level at baseline.

An advantage of HLM is that these predictors of level 1 cortisol functioning can become outcomes of interest using a slopes-as-outcomes approach (Snijders and Bosker, 1999). Individual differences in these terms were modeled to allow for each individual to have different cortisol levels at baseline (*U*_0_); different levels of cortisol down-regulation across the day (*U*_1_); or different rises in cortisol in response to waking (*U*_2_). The equations below illustrate that the CRHR1 genotype was entered as a main effect on cortisol level at baseline. Furthermore, perceived stress (*γ*_21__*PSS*_) and standard drinks consumed over the past 90 days (*γ*_22*EtOH*_) were entered as main effects on CAR.

$$\begin{array}{lll} \text{Level 1}\left(\text{within-individual}\right)\text{ Cortisol} & \text{=} & \beta_{\text{0}}+\beta_{\text{1}TSW}+\beta_{\text{2}CAR}+\text{r}\\ \text{Level 2}\left(\text{between individual}\right)\beta_{\text{0}} & \text{=} & \gamma_{00}+\gamma_{01CRHR1}+U_{0}\\ \beta_{\text{1}TSW} & \text{=} & \gamma_{10}+U_{1}\\ \beta_{\text{2}CAR} & \text{=} & \gamma_{20}+\gamma_{21PSS}+\gamma_{22EtOH}+U_{2} \end{array}$$

## Results

### Daily Stressors

Work (20.2%), Money (15.9%), and Neighborhood (15.5%) stress were identified as the top three stressors that were influencing one’s daily life. Work related stressors included problems finding a job, being laid off, and experiences of racism, discrimination, or some other type of unfair treatment. Money related stressors coalesced around difficulties making enough money, paying bills, and providing for one’s family. Finally, neighborhood stress was largely associated with living in poverty, fear for one’s safety, and exposure to drugs, crime, and violence.

### Stress and Alcohol Use

All of the participants reported that they experienced stress during the past month. More specifically, the scores on the PSS ranged from 8 to 40 with an average score of 24.7 (*SD* = 6.3). Additionally, 63.5% of the participants reported alcohol consumption during the past 90-days. On average, the participants reporting consuming approximately 14 drinks (*SD* = 28.7) during this reporting period (range: 0–140).

### Basal Cortisol Levels

TSW represented a significant linear slope from waking until the last sample was collected [*β*_1_ = –0.0003, *t*(70) = –5.71, *p* < 0.001]. On average, cortisol levels decreased throughout the day. CAR also represented a significant linear slope to the model [*β*_2_ = 0.07, *t*(70) = 3.56, *p* = 0.001]. This positive relationship indicated that cortisol levels increased from waking to approximately 30 min after waking. After accounting for the diurnal rhythm and the CAR, 70.6% of the total variance in cortisol was found to be a function of systematic individual differences [*χ*^2^(52) = 225.56, *p* < 0.001] and the remaining 29.4% of the variance in cortisol was derived from moment to moment fluctuations in cortisol beyond the diurnal rhythm or CAR. This suggests that rural African American emerging adults have moderately systematic salivary cortisol levels.

### Effect of CRHR1 Genotype on Cortisol Level at Baseline

**FIGURE 1:**
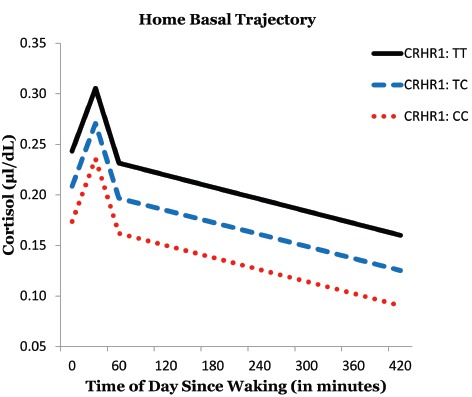
**Effects of CRHR1 genotype on basal cortisol levels**.

This sample had the following genotype frequencies: CC (38.8%), CT (40%), TT (7.1%); with 7.1% missing data. We tested to see if some of the variability in basal cortisol levels could be accounted for by an individual’s genotype. The T allele was coded to indicate greater dose: TT = 2, TC = 1, CC = 0. The CRHR1 SNP (rs4792887) had a significant linear relationship with cortisol level at baseline [*γ*_01_ = 0.048, *t*(69) = 2.21, *p* = 0.031]. Rural African American emerging adults with the TT genotype of the CRHR1 gene tended to produce the most basal cortisol throughout the day (Figure 1). Furthermore, participants with the CC genotype produced the least amount of basal cortisol. This provided some initial evidence for an additive relationship, with each additional T allele associated with greater basal cortisol output.

### Alcohol Consumption and Perceived Stress Differences in the CAR

**FIGURE 2:**
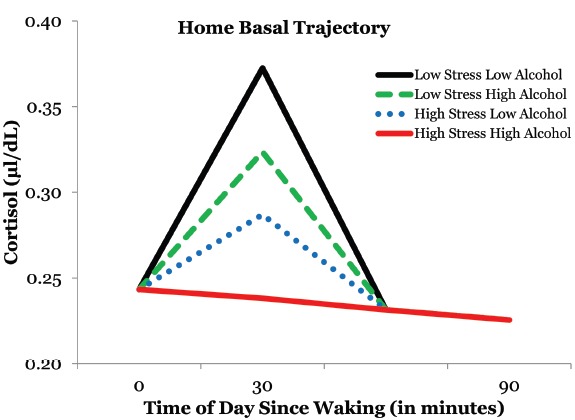
**The effects of alcohol consumption and perceived stress on the cortisol awaking response**.

We tested to see if some of the variability in CAR could be accounted for by perceived stress over the past month and standard drinks consumed over the past 90 days. Perceived stress had a significant inverse relationship with CAR [*γ*_21_ = –0.007, *t*(68) = –2.33, *p* = 0.023]. More specifically, rural African American emerging adults had a blunted CAR when experiencing higher levels of perceived stress. Furthermore, alcohol consumption also had a significant independent inverse relationship with CAR [*γ*_22_ = –0.003, *t*(68) = –2.13, *p* = 0.037] such that the CAR was blunted in individuals who consumed higher amounts of alcoholic beverages. Moreover, the CAR was essentially non-existent in participants who reported higher levels of stress and alcohol consumption (Figure 2). The interaction between reported stress and alcohol consumption was not a predictor of CAR suggesting additive effects of perceived stress and alcohol consumption. Additionally, there wasn’t a gender effect in predicting CAR.

## Discussion

The CAR has been identified as a reliable indicator of HPA-axis functioning (Chida and Steptoe, 2009; Gonzalez et al., 2009; Thorn et al., 2009). In this study, rural African American emerging adults exhibited a blunted CAR when reporting higher levels of stress or alcohol consumption. Moreover, these effects were additive, such that the CAR was obliterated for participants who reported both the experience of higher stress and increased alcohol consumption. While stress was only measured quantitatively over the past month, this outcome is consistent with previous studies that found a hypoactive CAR to be linked to chronic stress, fatigue, burnout, and hopelessness (Chida and Steptoe, 2009; Fries et al., 2009). This is further substantiated by the types of chronic stressors that were identified by the stress card sort. Furthermore, previous research linked alcohol abuse with hyperactive HPA activity (Adinoff et al., 2003). It is our hypothesis, that alcohol is being consumed as a negative coping strategy for addressing unmitigated stressors that this community endures on a daily basis. The long-term effect of heavy alcohol use will primarily mirror that of the stress model where acute alcohol consumption aimed at coping with daily life stress will initially lead to a hyperactive HPA-axis. However, the body’s chronic exposure to increased levels of alcohol will consequentially cause wear-and-tear on this regulatory system and ultimately result in a hypoactive HPA-axis due to allostatic load (Lovallo et al., 2000; Koob and Le Moal, 2001; McEwen, 2004; Lovallo, 2006). This is consistent with our finding that an increased level of alcohol consumption was linked to a blunted CAR.

Additionally, this study investigated the role that genotype—specifically CRHR1—might have in the production of basal cortisol. African American emerging adults with a CC genotype in the CRHR1 SNP (rs4792887) showed the lowest production of basal cortisol while participants with the TT genotype showed the highest levels of basal cortisol. Previous studies have identified the T allele as a risk factor in depression and suicidality (Wasserman et al., 2008, 2009). While exploratory, the data suggests an additive effect where the T allele in this particular SNP may be a risk factor associated with psychological distress which may lead to the production of higher basal cortisol. This is of particular importance given the growing body of literature linking dysregulated HPA-functioning with poor health outcomes (Fries et al., 2009).

It is important to note that experiences of stress related to work (i.e., finding a job, being laid off, experiences of racism, and discrimination), money (i.e., difficulty paying bills and providing for one’s family), and neighborhood disorganization (i.e., living in poverty, fear for one’s safety, and exposure to drugs, crime, and violence) were the top three chronic stressors identified by this sample of rurally situated African American emerging adults. This data is consistent with national trends showing African Americans (16.0%) experiencing an unemployment rate that nearly doubles that of their European American counterparts (8.7%; U.S. Department of Labor, 2011). More specifically, this cohort reported an unemployment rate of 63.1%. While it remains unclear if this cluster of chronic stressors is a function of race, rural status, age, and/or post-recession outcomes, there is little doubt that this community is exposed to unmitigated stressors that may have a deleterious effect on their long-term health outcomes. This “wear and tear” already appears evident within these emerging adults’ HPA axis.

While this study provides some unique data illustrating a relationship between stress, alcohol consumption, genotype, and HPA dysregulation with a rarely studied at-risk population, it is not without limitations. First, this study could benefit from a more nuanced assessment of environmental and neighborhood characteristics that serve as chronic stressors for this population. While the card-sort and assessment of perceived stress generated interesting data to substantiate the need for further investigation, more objective assessments utilizing geographic information system (GIS) mapping could accurately characterize drug availability, potential sources of chronic stress, and risk factors associated with drug use in the neighborhoods that the research participants currently reside in. Secondly, basal cortisol samples were collected at six points across the day; with the last sample being collected at approximately 4:30 pm. Given the termination of sample collection in the early evening, we are unable to model continued decreases in cortisol levels through bedtime. While this was done to time match these samples with a laboratory-based stress paradigm that took place in a second wave of this study, not reported here, the basal data utilized in the HLM model illustrates a clear reduction in cortisol across time, but doesn’t include the lowest point prior to sleep. Additionally, salivary cortisol was only collected across 1 day and did not include an objective assessment of time reporting. Therefore, there could be some error in the self-reporting of time in addition to the inability to make conclusions regarding the trait levels of basal cortisol due to the potential of day-to-day variations. Of note, we did not assess which participants from this community sample were currently enrolled in school. As a result, we could not assess what percentage of the unemployment rate was a function of being enrolled in school—regardless of work and money being identified as the top two stressors influencing their daily lives. Finally, this sample consisted of African American emerging adults residing in rural counties. Generalizing these findings to urban/suburban communities and/or settings should only be done with caution until such data becomes available. Given these limitations, the data provides some novel insights into the relationship between alcohol consumption, perceived stress, and CRHR1 genotype with the HPA-axis in a rarely studied sample of rural African Americans.

Future research could benefit from a prospective longitudinal research design that can ascertain if stress dysregulation leads to drug-use vulnerability, if drug-use accelerates the process of stress dysregulation, or both. Understanding the directionality of this relationship could lead to the identification of robust targets of intervention and prevention. This area of research could also be advanced by utilizing objective GIS data in characterizing neighborhood environments (Theall et al., 2012, 2013). Additionally, this body of literature could be enhanced by integrating decision-making variables, such as delayed discounting or alcohol demand, as proximal mechanisms that connect stress dysregulation to actual choices to drink or not to drink (MacKillop et al., 2010, 2011). Finally, the inclusion of objective measures of ethnicity and culture—in addition to other at-risk populations—could shed some light on within-group and between-group drug-related health disparities that disproportionately affect marginalized communities. In conclusion, the African American community disproportionately experiences poor health outcomes and further investigation is needed in this area to uncover and refine the mechanisms that can explain and eliminate this growing public health problem.

## Author Contributions

All authors contributed in a significant way to the manuscript and have read and approved the final manuscript.

### Conflict of Interest Statement

The authors declare that the research was conducted in the absence of any commercial or financial relationships that could be construed as a potential conflict of interest.
